# Child abuse during COVID-19 pandemic: what we can see

**DOI:** 10.1192/j.eurpsy.2022.362

**Published:** 2022-09-01

**Authors:** F. Reina, F. Vitrano

**Affiliations:** 1 Università degli studi di Palermo, U. O. Child Neuropsychiatry Unit, Policlinico Paolo Giaccone, Palermo, Italy; 2 ASP 6, Eiam, Palermo, Italy

**Keywords:** child welfare service, home isolation, Child abuse, Covid-19

## Abstract

**Introduction:**

COVID-19 caused an ongoing health emergency that rapidly spread worldwide, so all countries adopted exceptional health measures to reduce disease’s transmission. The stress caused by pandemic presents increasing risks for family violence and for child abuses. Interinstitutional Equips of Abusive and Maltreatment (IEAM) deals with the management of abusive families in Palermo’s territory. IEAM starts evaluations after the interventions of the court solicited by a complaint filed by teachers, law enforcements or members of the family. IEAM is formed by consultations in maternity ward, child welfare service and school educational psychologists.

**Objectives:**

The purpose of this research was to evaluate the variations of child abuse and maltreatment reported during local and National lockdown due to pandemic. Reported cases were compared with the previous year.

**Methods:**

The authors collected data of IEAM’s advisory from January 2019 until August 2021. The number of cases was evaluated monthly.

**Results:**

We observed 124 cases in 2019, 145 in 2020 and 94 until August 2021. Advisory reductions coincided with the service activity reduction in August of every year and in March 2020 when Italy declared national lockdown. Social isolation represents a risk factor for child abuse. Although the increase of cases was quite stable, there are reasons to speculate that the reporting of child abuse and maltreatment decreased since home isolation hampered the access to responsible services.

**Conclusions:**

School closure together with the strong reduction of social care and monitoring during and after lockdown might have increased the domestic violence. Lastly, the child abuse may be underreported despite the effective increase.

**Disclosure:**

No significant relationships.

